# Age-group differences in trust-related decision-making and learning

**DOI:** 10.1038/s41598-023-50500-x

**Published:** 2024-01-02

**Authors:** Marilyn Horta, Alayna Shoenfelt, Nichole R. Lighthall, Eliany Perez, Ian Frazier, Amber Heemskerk, Tian Lin, Robert C. Wilson, Natalie C. Ebner

**Affiliations:** 1 Department of Psychology, University of Florida, Gainesville, FL USA; 2 Pain Research and Intervention Center of Excellence, University of Florida, Gainesville, FL USA; 3 Department of Psychology, University of Central Florida, Orlando, FL USA; 4 Graduate School of Applied and Professional Psychology, Rutgers University, New Brunswick, NJ USA; 5 Department of Psychology, University of Arizona, Tucson, AZ USA

**Keywords:** Human behaviour, Psychology

## Abstract

Facial impressions contribute to evaluations of trustworthiness. Older adults are especially vulnerable to trust violations, incurring risks for deception and exploitation. Using the newly developed social Iowa Gambling Task (S-IGT), we examined age-group differences in the impact of facial trustworthiness on decision-making and learning. In the congruent condition (CS-IGT), advantageous decks were paired with trustworthy faces and disadvantageous decks with untrustworthy faces. In the incongruent condition (IS-IGT), this pairing was reversed. Younger (n = 143) and older (n = 129) participants completed either the standard Iowa Gambling Task (IGT), CS-IGT, or IS-IGT. Both age groups preferred trustworthy faces in their initial choices. Older adults performed worse than younger adults across all tasks over time. Further, compared to younger adults, older adults performed worse on the IS-IGT, suggesting that incongruent facial cues interfered with older adults’ performance, which aligns with reduced sensitivity to negative social reputations in aging. Multilevel modeling also indicated that age-group differences were most pronounced across all tasks in the last 40 trials. Together these findings suggest that differences between younger and older adults in experience-dependent decision-making are magnified in social contexts that involve a “wolf in sheep’s clothing,” which may reflect age-related difficulties in integrating incongruent information.

## Introduction

Trust is important for healthy interpersonal exchanges and relationships^1,2^. Evaluations of trustworthiness, however, are sometimes inaccurate and expectations of trust can be violated with negative social and economic consequences. Older adults experience an increased risk of financial exploitation^3^, including the prevalent, yet still underestimated, instances of fraud and scams targeting this age group^4^. Thus, the ability to discern trustworthiness in others is a critical skill to be preserved across adulthood and aging.

Perceiving trustworthiness is a complex and dynamic social-cognitive process that is influenced by both immediate first impressions as well as deliberative evaluations of others that evolve as additional reliable information and experience are acquired^2,5–7^. First impressions of trustworthiness can be quickly inferred from faces^8–11^. However, first, immediate impressions from faces are not necessarily accurate^12^. Impressions of trustworthiness from faces are highly related to other perceptual characteristics, including facial attractiveness^9^, emotional expression^9,13^, age^14^, and self-resemblance^15,16^; and can be unreliable^17^, susceptible to bias^9,18^, and overweighed in social decisions^19^. For example, overreliance on first impressions of trustworthiness may lead to negative social attributions and behaviors towards others with possibly devastating consequences (e.g., dehumanization of outgroup members^20^). Thus, facial cues that initially influence perceptions of trustworthiness do not constitute sufficient information to accurately determine the trustworthiness of others, especially long term^19^.

Therefore, updating first impressions of trustworthiness by integrating more reliable information over time, such as behavioral feedback and prior experience, is critical for evaluating others’ trustworthiness in a way that minimizes risk and maximizes positive social decision-making outcomes (e.g., reciprocity, cooperation)^17^. Despite the importance of this topic, there is a lack of clarity on the impacts of facial trustworthiness on social decision-making initially, over repeated interactions, and in the context of aging.

Evaluations of trustworthiness are subject to change across the lifespan, but the characterization of age-related changes is still limited^14,17^. Preferences for trustworthy faces emerge as early as 6 months old^21^. There is also general agreement between younger and older adults on their ratings of trustworthiness for trustworthy-looking faces based on first impressions^14,22–25^, indicating that first impression accuracy of trustworthy faces may not be impaired in older age^26,27^. However, younger and older adults appear to differ in the magnitude of this effect in that older adults tend to evaluate faces more positively than younger adults overall^27–29^, with this age effect most notable for faces associated with untrustworthy cues, including disreputable backgrounds and behavior^24,28–31^. Although older compared to younger adults show less distinctive trustworthiness ratings for untrustworthy-looking faces, prior work shows that they have similar behavioral preferences for trustworthy over untrustworthy cues in choice tasks^25,31,32^.

Increased age-related difficulties identifying emotions^33^ and deception^34^ from faces may impact the accuracy of trustworthiness judgments^26^. Differences in trustworthiness evaluations between younger and older adults may also be due to motivational changes such as the positivity effect, which involves diminished processing of negative, relative to positive, information^27–29^, including dampened signaling for loss-predictive cues^24,35^. It is crucial to improve current understanding of age-similarities vs. age-differences in trust-related decision-making and learning to address the unique vulnerabilities older adults face in social situations that require accurate trustworthiness judgements.

Economic games provide a foundation to behaviorally assess social decision-making^2^. However, much work has either utilized economic games with one-shot designs, in which participants interact with a social partner once, or limited trials (e.g., Trust Game) which do not reflect the complexity of long-term, repeated interactions with social partners. The use of tasks with many trials and more complex outcomes that reflect costs and benefits of a choice may be more relevant to capturing real-world trust-related decision-making. That is, trustworthiness evaluations are updated over time as familiarity with social partners increases and may have special relevance for behavior that contributes to susceptibility to deception and fraud among older adults^4^. There is currently limited work utilizing multi-round economic games in older populations. Of the existing studies, some indicate that compared with younger adults, older adults are more trusting of untrustworthy partners in these games^36^ and exhibit less learning about trustworthiness^31,36–38^. Also, vigilance for subtle cues of untrustworthiness and minor transgressions is reduced among older adults^38^. Further, overreliance on information conveyed by facial cues among older adults may result in greater persistence of suboptimal choices in repeated social exchanges^31^.

One economic game that is widely used in cognitive science and was initially designed to represent the uncertainty of reward and risk of punishment in real-world decision-making, is the Iowa Gambling Task (IGT)^39,40^. In this task, participants are instructed to repeatedly draw cards from decks with varying rewards and punishments to maximize points. Over time, participants can learn which decks are more advantageous to choose from than others in part due to the affective signals this task evokes in response to feedback^40^. The IGT has been widely implemented in healthy and clinical populations, including studies of older adults^41–44^. Although there is evidence for age-related impairments in performance when compared to younger individuals^43,44^, some older adults are relatively more adaptive decision-makers than other older adults in this task^41,42,45^. Older adults are also better able to understand task contingencies later in the IGT as experience accumulates over time^42^.

**Figure 1:**
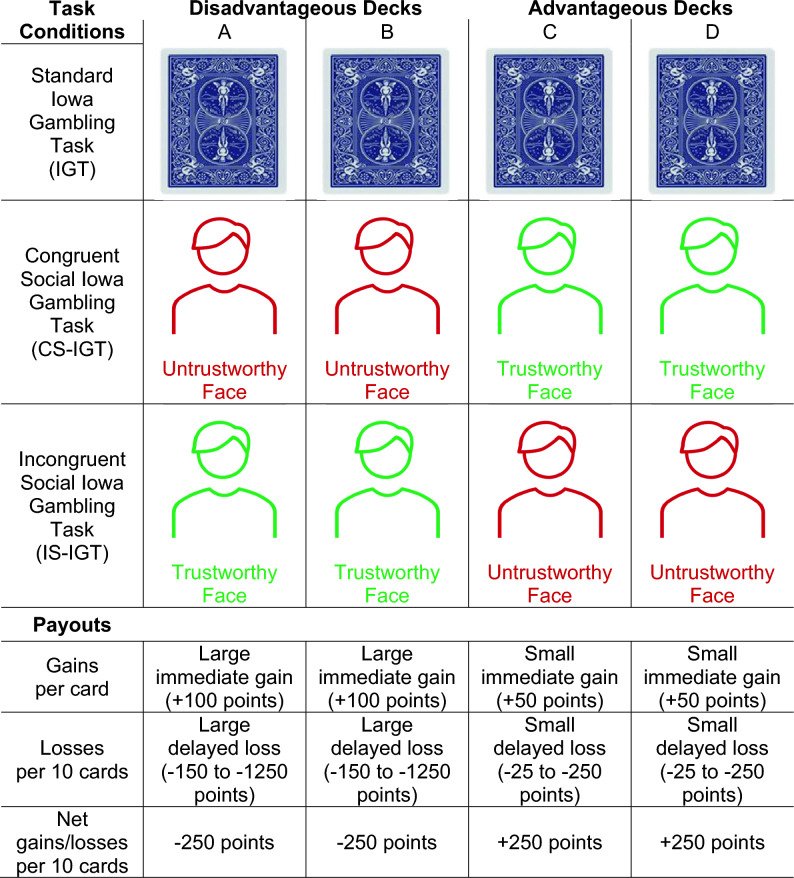
Task conditions and design features. There were three task conditions: Standard Iowa Gambling Task (IGT), Congruent Social Iowa Gambling Task (CS-IGT), and Incongruent Social Iowa Gambling Task (IS-IGT). In the CS-IGT, advantageous decks were paired with naturalistic photographs of trustworthy faces (placeholder depicted in green) and disadvantageous decks with untrustworthy faces (placeholder depicted in red). These photographs (not pictured here) were from the FACES database^46^. In the IS-IGT, this pairing was reversed. In each task condition, participants started with 2000 points and drew a total of 100 cards from 4 decks. For every card drawn, participants received feedback on how many points they won (i.e., + 50 points for an advantageous deck draw (**C** or **D**) and + 100 points for a disadvantageous deck draw (**A** or **B**)). Occasionally (i.e., approximately every 10 cards), participants also received feedback of a penalty loss of points (i.e., between − 25 and − 250 points for advantageous deck draws and between − 150 and − 1250 points for disadvantageous deck draws). Performance was calculated as the total number of draws from advantageous decks minus the total number of draws from disadvantageous decks with larger, positive performance scores indicating better decision-making and smaller, negative performance scores indicating poorer decision-making. Only performance from the first four of the five blocks was examined due to task design, in which participants drew from non-replenishing card decks and due to a few participants running out of cards by the end of the fourth block (see Supplementary Information).

The current study used the standard IGT as well as a newly developed social variant of the IGT (S-IGT) to characterize age-group differences in trust-related decision-making and learning. One advantage of the S-IGT is that it follows the same design and reinforcement schedule as the well-established IGT (Fig. 1). This approach allows for a direct comparison of findings from the present study with the standard IGT used in previous studies and as shown in this paper for the first time, provides a non-social control condition crucial for delineating the effects on learning unique to social cues (i.e., facial trustworthiness) in younger and older adults.

The S-IGT expands on the standard IGT’s extended, iterative, and learning-dependent decision-making environment by adding social stimuli that may influence choices via the presentation of faces that vary in perceived trustworthiness^14,46^. Similar experimental manipulations have been achieved in previous studies in which the incorporation of certain images resulted in avoidant behaviors among anxious individuals (e.g., spider-fearful individuals avoided decks with images of spiders^47^; socially anxious individuals avoided decks with angry faces^48^). These studies effectively demonstrated that decision-making can be altered by incorporating social-affective visual cues to the IGT design and can vary according to individual differences (e.g., anxiousness).

The S-IGT specifically assessed the impact of facial cues that are congruently (i.e., CS-IGT) or incongruently (i.e., IS-IGT) paired with advantageous and disadvantageous outcomes on trust-related decision-making and learning. Congruent and incongruent facial cues can implicitly enhance or interfere with trust-related judgments in repeated interactions^5,49^. Furthermore, the inclusion of faces that vary in facial trustworthiness provides the unique opportunity to determine the extent to which facial biases affect initial choice as well as learning over time, where initial biases must be overridden, and decision-making strategies updated, to achieve the goal of gaining maximal points. Thus, our social cue manipulation contributes additional real-world value to the IGT paradigm in a way that is highly relevant for studying older adults, who are more vulnerable when navigating social situations where incongruent information is subtly or implicitly presented, such as in the case of susceptibility to scams^4^ or interpersonal deception^34^.

Based on the consensus between age groups on first impressions of trustworthy over untrustworthy faces^25,31^, we predicted that facial trustworthiness would impact first deck choices, and comparably so in both age groups. Specifically, decks with trustworthy faces would be more frequently chosen at first by both younger and older adults (*Hypothesis 1*).

To replicate previous evidence of age-related differences in overall performance during the standard IGT^43,44^, we hypothesized that younger adults would perform better overall on the IGT than older adults (*Hypothesis 2a*). Furthermore, building on previous results that younger and older adults are comparable in their evaluations of trustworthy faces and at integrating congruent information^31,32^, we predicted that both age groups would perform comparably on the CS-IGT overall (*Hypothesis 2b*). However, given that incongruent contexts interfere with older adults’ social-cognitive abilities (e.g., detecting deception^34^, disambiguating facial expressions^50^) and decision-making^31^, we expected that younger adults would perform better overall on the IS-IGT than older adults (*Hypothesis 2c*).

In addition, given well-established age-related declines in learning in the IGT^43,44^, we hypothesized that, compared to younger adults, older adults would show less improvement in performance over task blocks (i.e., less learning) across the IGT, CS-IGT, and IS-IGT (*Hypothesis 3a*). We also expected that these age-group differences in learning over time would be attenuated in the CS-IGT, but not the IS-IGT (*Hypothesis 3b*), given that older adults tend to rely more on congruent social information and judge negative behavior of others less than younger adults^31,51^.

Learning follows a “continuum of certainty”^52^, where an individual's initial behavior in a decision-making task will reflect the exploration of available options until enough experience with task contingencies is accumulated to form preferences for certain options. These preferences may result as a function of monitoring desirable and undesirable outcomes over time (e.g., IGT) and may also be influenced by the inclusion of additional cues (e.g., faces^48^). There are no well-defined boundaries between the transition from uncertainty to certainty in learning between individuals^52^. However, when compared to healthy controls, alcohol-dependent individuals were found to have lower performance in the IGT, especially in the latter half of the task as evidenced by riskier choices^53–55^. Additionally, and of particular relevance here, meta-analytic evidence supports that older adults do not discriminate between advantageous and disadvantageous decks in the IGT until the latter half of the task as marked by a strong reversal learning effect starting in Block 3^42^. It is unknown, however, if decision-making and learning differ across the earlier or later phases of the S-IGT (i.e., under uncertainty or experience with task contingencies) for younger and older adults.

We expected that the inclusion of visual cues (i.e., faces) would promote better performance in the earlier half of the S-IGT (i.e., uncertainty phase, first 40 trials) and therefore earlier learning of task contingencies compared to the standard IGT. We hypothesized that older adults would have similar performance as younger adults in the uncertainty phase (i.e., first 40 trials) but worse performance than younger adults in the experience phase (i.e., last 40 trials) across all task conditions (*Hypothesis 4a*). We also expected, however, that the inclusion of congruent information in the CS-IGT would result in an earlier understanding of task contingencies (i.e., choosing trustworthy faces leads to advantageous outcomes and choosing untrustworthy faces leads to disadvantageous outcomes) and therefore better performance in the uncertainty phase of the CS-IGT than in the IS-IGT and the IGT for both age groups (*Hypothesis 4b*). Concurrently, because incongruent information from the IS-IGT could yield stronger error signals due to a mismatch in expectations and outcomes early in the task (i.e., choosing trustworthy faces leads to disadvantageous outcomes, and choosing untrustworthy faces leads to advantageous outcomes), we predicted better performance in the uncertainty phase of the IS-IGT than the IGT, but not the CS-IGT, for both age groups (*Hypothesis 4c*).

## Methods

### Participants

The present investigation used data from two studies (referred to here as Study 1 and Study 2) that were each approved by the University of Florida Institutional Review Board. All methods were performed in accordance with relevant guidelines and regulations. Study 1 comprised a total of 65 younger (M = 21.11 years old, SD = 2.51; Range = 18–33 years old; 53.85% women) and 67 older (M = 75.21 years old; SD = 6.28; Range = 64–96 years old; 52.24% women) adults. Study 2 comprised 78 younger (M = 22.14 years old; SD = 3.87; Range = 18–35 years old; 84.62% women) and 62 older adults (M = 69.76 years old; SD = 6.08; Range = 61–82 years old; 66.13% women). Three participants from Study 1 were excluded due to missing or incomplete data, resulting in a final sample across both studies of 143 younger (M = 21.67 years old; SD = 3.35; Range = 18–35 years old; 70.63% women) and 129 older (M = 72.59 years old; SD = 6.74; Range = 61–96 years old; 58.91% women) adults for this report.

Table 1 presents sample demographic information. Years of formal educational attainment were significantly greater in older (M = 16.31 years) than younger adults (M = 15.03 years) (F[1, 261] = 14.65, *p* < 0.001), but there was no significant difference in years of education between individuals across task conditions (i.e., IGT, CS-IGT, IS-IGT). Also, neither chronological age (F[2, 269]  = 1.42, *p* = 0.24) nor gender (i.e., men vs. women; χ^2^(2) = 2.19, *p* = 0.33) distributions significantly differed across task conditions.

**Table 1:** Sample demographics for the total sample (N = 272) by Age group and Task condition.

	IGT		CS-IGT		IS-IGT	
	Younger (n = 46)	Older (n = 29)	Younger (n = 55)	Older (n = 51)	Younger (n = 42)	Older (n = 49)
Chronological age M (SD)	22.02 (3.84)	72.86 (6.39)	22.00 (3.83)	73.14 (7.61)	20.86 (1.57)	71.96 (6.02)
Gender % Women	80.43%	58.62%	67.27%	56.86%	64.29%	61.22%
Years of education M (SD)	15.11 (2.38)	16.52 (2.09)	15.12 (1.94)	16.41 (4.26)	14.87 (1.35)	15.99 (2.51)

There was no significant effect of Task condition for any of these demographic variables at *p* < 0.050. Education was measured by total years of formal education, which was unavailable for five participants (i.e., 1 older adult in the IGT, 2 younger adults in the CS-IGT, and 2 older adults in the IS-IGT).

IGT, Iowa Gambling Task; CS-IGT, Congruent Social Iowa Gambling Task; IS-IGT, Incongruent Social Iowa Gambling Task; M, Mean; SD, Standard deviation.

### Procedure

All participants provided written informed consent. Participants were recruited from existing university participant registries (e.g., the SONA system participant pool for younger adults and an internal registry for older adults) and throughout the community via fliers, mailouts, radio and newspaper ads, and word-of-mouth. Adopting a between-subjects design, participants were pseudo-randomly assigned, in an age-by-gender matched fashion, to only one of the three task conditions: IGT, CS-IGT, or IS-IGT (described below) to avoid practice effects^56,57^. Study sessions took place in the lab and lasted about 1 h for Study 1 and 2 h for Study 2. The sessions closed with debriefing and compensation.

Participants recruited through the SONA pool (n = 10 in Study 1, n = 0 in Study 2) received 3 research credits as base compensation; all other participants (n = 122 in Study 1; n = 140 in Study 2) received $20 as base compensation. All participants received a bonus of up to $10 depending on their task performance to ensure that deck draws had real economic outcomes for participants, which should have motivated them to draw from decks that yield more points over time^58^. SONA vs. non-SONA participants showed comparable results.

### The standard and social Iowa Gambling Tasks

The standard IGT refers to the non-social control task condition in which all decks are represented by a Bicycle brand card design. The CS-IGT refers to the congruent social task condition in which advantageous decks are paired with trustworthy faces and disadvantageous decks are paired with untrustworthy faces. The IS-IGT refers to the incongruent social task condition in which advantageous decks are paired with untrustworthy faces and disadvantageous decks are paired with trustworthy faces. See Fig. 1 for a depiction of the task conditions and their design features.

The faces used in the CS-IGT and IS-IGT were independently rated as the two most trustworthy and two least trustworthy white, male, middle-aged faces with a neutral (non-emotional) expression in the FACES database^14,46^. Middle-aged faces were selected to avoid own-age bias in trust perception^30,59^ and neutral expressions were chosen to avoid emotion-related bias (i.e., negative emotions can be perceived as less trustworthy^14^). However, sex- and race-associated facial features were not manipulated in the present study.

Each task condition was programmed in PsychoPy3 v3.0.6^60^ with the same reinforcement schedule as the IGT as described by Bechara and colleagues^39,40^ and administered on a lab computer. In each task condition, participants started with 2000 points and drew a total of 100 cards from 4 decks with the goal of maximizing points. Participants were informed that some decks were more advantageous than others but were not told which nor were they told how many draws needed to be made. Each deck had a total of 40 non-replenishing cards. The computer screen included the point total for the participant’s current and previous turn. Once all the cards of a particular deck were drawn, a red “X” would appear in place of the deck to indicate that the deck was depleted and to prompt participants to draw from other decks. All task conditions were self-paced.

In all task conditions, as in the original IGT^39^, two of the decks were designed to be “disadvantageous” (i.e., resulting in a net loss of 250 points per 10 cards) and the other two were “advantageous” (i.e., resulting in a net gain of 250 points per 10 cards). For every card drawn, participants received feedback on how many points they won (i.e., + 50 points for an advantageous deck draw [C or D] and + 100 points for a disadvantageous deck draw [A or B]). Occasionally (i.e., approximately every 10 cards), participants also received feedback of a penalty loss of points (i.e., between − 25 and − 250 points for advantageous deck draws and between − 150 and − 1250 points for disadvantageous deck draws). Cards drawn from advantageous decks resulted in immediate small gains and delayed small losses. Cards drawn from disadvantageous decks resulted in large immediate gains and large delayed losses.

Both disadvantageous decks (A and B) were always located on the left-hand side of the screen and both advantageous decks (C and D) were always located on the right. The two faces associated with specific advantageous and disadvantageous decks, respectively, were counterbalanced in the CS-IGT and IS-IGT. Thus, participants were pseudo-randomly assigned to one of the five possible task versions.

### Statistical analyses

No outliers were determined that exceeded + /− 3 SD from the mean total performance score for each age group across all blocks of each task condition. When determining outliers within each of the blocks, four performance scores were identified as outliers for three unique participants (i.e., two younger participants and one older participant). Removing these outliers did not change the results and therefore were retained in this report. Also, no participant showed invalid performance via exclusively choosing from advantageous decks^61^ (see Supplementary Information).

See Supplementary Table 1 for an overview of the hypotheses as well as the design and factors associated with each statistical analysis described next. To first determine if participants were more likely to make their initial choice from a deck represented by a trustworthy, over an untrustworthy, face (*Hypothesis 1*), we conducted a one-sample Chi-Square Test on participants’ first deck choice in the S-IGT. We then followed up with a Cochran-Mantel–Haenszel test to examine if this effect was comparable in the two age groups. The outcome variable for the Chi-square and Cochran-Mantel–Haenszel tests on first deck choice was derived from binary values representing participants’ first choice as a function of the face presented (1 = trustworthy face, 0 = untrustworthy face). For the Chi-Square test, the total sum of first choices for decks represented by either trustworthy or untrustworthy faces was then divided by the total first choice data points available (i.e., 197), converting first choices into percentages. This analysis used the following design: 2 Face (trustworthy vs. untrustworthy), controlling for Data source (Study 1 vs. Study 2). Accordingly, for the Cochran-Mantel–Haenszel test, the total sum of first choices for decks represented by either trustworthy or untrustworthy faces for both younger and older adults was then divided by the total first choice data points available (i.e., 97 for younger adults and 100 for older adults), converting first choices into percentages. This analysis used the following design: 2 Age group (younger vs. older) × 2 Face (trustworthy vs. untrustworthy), controlling for Data source (Study 1 vs. Study 2).

Next, we predicted age-group differences in performance in the IGT (*Hypothesis 2a*), age-group similarities in performance for the CS-IGT (*Hypothesis 2b*), and age-group differences in performance for the IS-IGT (*Hypothesis 2c*). We also expected less improvement over time in all task conditions for older compared to younger adults (*Hypothesis 3a),* with this effect attenuated in the CS-IGT *(Hypothesis 3b*). To accommodate for the nested data structure of blocks within participants, these hypotheses were addressed using multilevel modeling (MLM)^62^. MLM is a flexible approach that considers differences in decision-making between and within individuals (e.g., Age group, Block) and task conditions (e.g., IGT vs. CS-IGT vs. IS-IGT) as well as their interactions^63^. The outcome variable was performance, which was calculated as the total number of draws from advantageous decks minus the total number of draws from disadvantageous decks, across four 20-trial blocks. Only performance from the first four of the five blocks was examined due to the present study’s task design, in which participants drew from non-replenishing card decks and due to a few participants running out of cards by the end of the fourth block (See Supplementary Information). The range for performance scores was − 20 to + 20 per block. Age group and Task condition were between-subjects independent variables and Block was a within-subject independent variable. This analysis used the following design: 2 Age group (younger vs. older) × 3 Task condition (IGT vs. CS-IGT vs. IS-IGT) × 4 Block (four 20-trial blocks), controlling for Data source (Study 1 vs. Study 2).

Finally, to examine differences in performance under uncertainty vs. experience across all task conditions by age group (*Hypotheses 4a, 4b, and 4c*), we conducted a separate MLM analysis on performance with Age group and Task condition as between-subjects independent variables and Choice phase (i.e., first 40 trials [uncertainty], last 40 trials [experience]) as a within-subject independent variable. This analysis used the following design: 2 Age group (younger vs. older) × 3 Task condition (IGT vs. CS-IGT vs. IS-IGT) × 2 Choice phase (first 40 trials [uncertainty] vs. last 40 trials [experience]), controlling for Data source (Study 1 vs. Study 2). Data source did not affect task performance in either of the two MLMs (i.e., Wald χ^2^(1) = 0.59, *p* = 0.44 and Wald χ^2^(1) = 0.76, *p* = 0.38, respectively).

## Results

### First deck choice in the S-IGT (Hypothesis 1)

The effect for first deck choice was significant (χ^2^(1,197) = 71.88, *p* < 0.001). As predicted, all participants were more likely to initially choose decks paired with trustworthy than untrustworthy faces (i.e., 80.2% of first choices were for a deck with a trustworthy face compared to 19.8% of first choices for a deck with an untrustworthy face). Furthermore, a non-significant Cochran-Mantel–Haenszel test (χ^2^(1,197) = 2.45, *p* = 0.12) found younger and older adults comparably first chose decks with trustworthy over untrustworthy faces in the S-IGT, with 75.3% of initial choices for a trustworthy face compared to 24.7% of initial choices for an untrustworthy face in younger adults vs. 85% of initial choices for a trustworthy face compared to 15% of initial choices for an untrustworthy face in older adults. Taken together, these findings supported *Hypothesis 1* in that there was an initial preference for trustworthy over untrustworthy faces in both age groups.

### Full-factorial multilevel model of effects for Age group, Task condition, and Block (Hypotheses 2a, 2b, 2c, 3a, 3b)

The interaction between Age group and Task condition for overall performance was significant (Wald’s χ^2^(2) = 10.68; *p* = 0.005). Pairwise comparisons indicated that younger, compared to older, adults performed better overall on the IGT (z = 3.29, *p* = 0.001) and the IS-IGT (z = 4.15, *p* < 0.001). However, overall performance was comparable between the two age groups in the CS-IGT (z = − 0.68, *p* = 0.50). Together, these findings supported *Hypotheses 2a*, *2b*, and *2c* that while there were no significant age-group differences in overall performance for the CS-IGT, younger adults significantly outperformed older adults in the IGT and the IS-IGT.

There was also a significant interaction between Age group and Block (Wald’s χ^2^(3) = 9.62; *p* = 0.02). Post-hoc examination of age-group differences by Block showed that across all task conditions, younger and older adults performed comparably during Block 1 (z = 0.41, *p* = 0.68), but younger adults significantly outperformed older adults in Block 2 (z = 2.17, *p* = 0.03), Block 3 (z = 3.57, *p* < 0.01), and Block 4 (z = 3.54, *p* < 0.01). This pattern of findings provides support for *Hypothesis 3a* in that by Block 2 the age groups significantly differed in learning marked by less improvement in performance across all tasks up to Block 4 among older, relative to younger, adults.

In addition, the interaction between Task condition and Block was significant (Wald’s χ^2^(6) = 24.14; *p* < 0.001). Post-hoc examinations revealed that Block 1 performance was significantly lower in the IS-IGT than the CS-IGT (z = − 4.52, *p* < 0.001) and the IGT (z = − 2.53, *p* = 0.01) across all participants. These findings indicate that facial cues of trustworthiness provided an initial hindrance in the IS-IGT. Block 4 performance was highest in the IGT compared to the CS-IGT (z = 2.72, *p* < 0.01) and the IS-IGT (z = 2.27, *p* = 0.02). Block 4 performance was comparable between the CS-IGT (M = 3.11; SD = 2.38) and the IS-IGT (M = 3.36, SD = 3.06; z = 0.40, *p* = 0.69). These results suggest that, over time, participants in the IS-IGT learned to ignore the facial cues—reaching performance levels of those in the CS-IGT. However, learning was greatest in the IGT which did not include additional congruent or incongruent facial cues to process.

**Figure 2:**
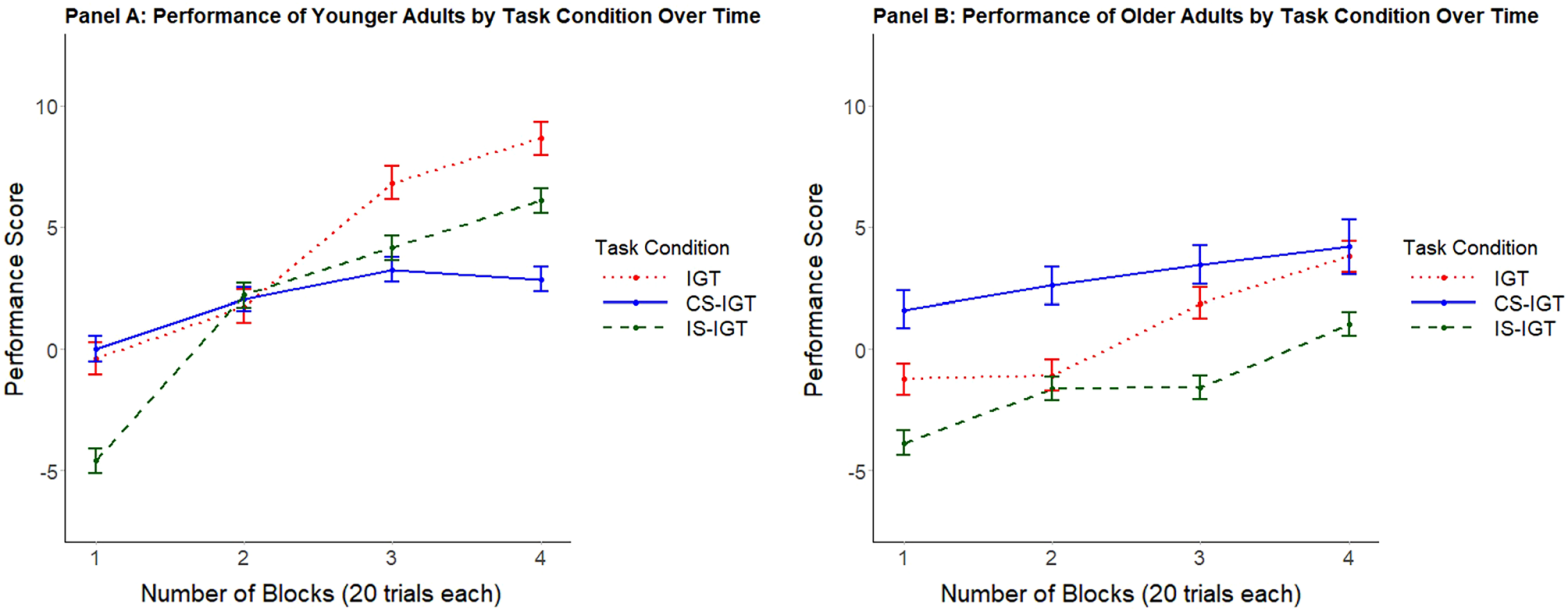
Task performance for (**A**) younger and (**B**) older adults in the standard Iowa Gambling Task (IGT; dotted red line), congruent social Iowa Gambling Task (CS-IGT; solid blue line), and incongruent social Iowa Gambling Task (IS-IGT; dashed green line). The x-axis indicates 4 blocks of 20 card draws each. The y-axis indicates performance score as the number of draws from advantageous decks minus the total number of draws from disadvantageous decks. Lines depict raw means and error bars depict 95% confidence intervals.

Notably, the interaction of Task condition and Block did not vary by Age group (Wald’s χ^2^(6) = 3.82; *p* = 0.701) (Fig. 2). Thus, both age groups initially performed worse in the IS-IGT than the CS-IGT and the IGT, yet older adults performed worse in all tasks over time. These results provide support for *Hypothesis 3a,* but not *Hypothesis 3b*, in that older adults showed impaired performance over time, but this effect was comparable across all tasks. See Table 2 for an overview of mean task performance by Age group, Task condition, and Block and Supplementary Table 2 for an overview of all significant and non-significant effects.

### Full-factorial multilevel model of effects for Age group, Task condition, and Choice phase (Hypotheses 4a, 4b, 4c)

The interaction between Age group and Task condition for overall performance was significant (Wald’s χ^2^(2) = 10.75; *p* = 0.005), consistent with findings in the previous model. The interaction between Age group and Choice phase was significant (Wald’s χ^2^(1) = 6.75; *p* = 0.009). Follow-up tests showed that younger and older adults did not differ in performance during the uncertainty phase (z = − 1.10; *p* = 0.27), but older adults performed significantly worse than younger adults during the experience phase (z = − 4.40, *p* < 0.001). These results provide support for *Hypothesis 4a* in that while there were age-group similarities in performance in the uncertainty phase, older adults showed worse performance in the experience phase across all task conditions.

Further, the interaction between Task condition and Choice phase was significant (Wald’s χ^2^(2) = 13.13; *p* = 0.001), in that performance across all participants in the CS-IGT was significantly better than performance in the IS-IGT (z = 4.07, *p* < 0.001) during the uncertainty phase. No other comparisons between task conditions in the uncertainty phase were significant. Though performance in the uncertainty phase for the CS-IGT was numerically higher than performance in the IGT, this comparison was not significant (z = 1.94, *p* = 0.052). Additionally, though performance in the uncertainty phase for the IS-IGT was numerically lower than performance in the IGT, this comparison was not significant (z = 1.85, *p* = 0.064). Together these findings partially support *Hypotheses 4b* and *4c*, indicating that a bias towards trustworthy faces facilitated better performance in the CS-IGT over the IS-IGT, but not the IGT, in the first 40 trials. Results reflect that the inclusion of both congruent and incongruent cues to the IGT design did not significantly improve performance in the uncertainty phase when compared to the standard IGT. However, congruent cues were more beneficial than incongruent cues during the uncertainty phase.

During the experience phase, participants performed significantly better in the IGT compared to the IS-IGT (z = 2.90, *p* < 0.01) and the CS-IGT (z = 2.05, *p* = 0.04). Participants generally showed improvement from the uncertainty phase to the experience phase across all task conditions, but this effect was especially pronounced in the IGT (z = 5.88, *p* < 0.001), followed by the IS-IGT (z = 4.85, *p* < 0.001), and then the CS-IGT (z = 2.53, *p* = 0.01). See Table 2 for an overview of mean task performance by Age group, Task condition, and Choice phase and Supplementary Table 3 for an overview of all significant and non-significant effects.

**Table 2:** Task performance: means (standard deviations) [95% confidence intervals] by Age group, Task condition, Block, and Choice phase.

	IGT		CS-IGT		IS-IGT	
	Younger	Older	Younger	Older	Younger	Older
Block 1	− 0.40 (2.28) [− 1.07, 0.29]	− 1.24 (1.69) [− 1.88, − 0.60]	0 (1.87) [− 0.51, 0.51]	1.61 (2.82) [0.81, 2.40]	− 4.62 (1.65) [− 5.13, − 4.10]	− 3.88 (1.75) [− 4.38, − 3.38]
Block 2	1.74 (2.28) [1.06, 2.42]	− 1.10 (1.69) [− 1.75, − 0.46]	2.04 (1.87) [1.53, 2.54]	2.59 (2.82) [1.79, 3.38]	2.19 (1.65) [1.68, 2.71]	− 1.63 (1.75) [− 2.13, − 1.13]
Block 3	6.83 (2.28) [6.15, 7.50]	1.86 (1.69) [1.22, 2.51]	3.24 (1.87) [2.73, 3.74]	3.45 (2.82) [2.66, 4.24]	4.14 (1.65) [3.63, 4.66]	− 1.59 (1.75) [− 2.09, − 1.09]
Block 4	8.65 (2.28) [7.97, 9.33]	3.79 (1.69) [3.15, 4.44]	2.84 (1.87) [2.33, 3.34]	3.41 (2.82) [2.62, 4.21]	6.10 (1.65) [5.58, 6.61]	1.02 (1.75) [0.52, 1.52]
Uncertainty phase (first 40 trials)	1.35 (3.28) [0.37, 2.32]	− 2.34 (2.38) [− 3.25, − 1.44]	2.04 (2.68) [1.31, 2.76]	4.20 (4.02) [3.06, 5.33]	− 2.43 (2.35) [− 3.16, − 1.70]	− 5.51 (2.50) [− 6.23, − 4.79]
Experience phase (last 40 trials)	15.48 (3.28) [14.50, 16.45]	5.66 (2.38) [4.75, 6.56]	6.07 (2.68) [5.35, 6.80]	6.86 (4.02) [5.73, 7.99]	10.24 (2.35) [9.50, 10.97]	− 0.57 (2.50) [− 1.29, 0.15]

IGT, Iowa Gambling Task; CS-IGT, Congruent Social Iowa Gambling Task; IS-IGT, Incongruent Social Iowa Gambling Task.

Together these findings indicate an age-group invariant pattern of learning in which experience-dependent performance gains were greatest when facial cues of trustworthiness were absent (in the IGT), then in the IS-IGT, and finally the CS-IGT. Greater learning in the IS-IGT was due to an initial bias toward disadvantageous deck draws followed by a relatively strong shift toward advantageous decks. Improvements in performance from earlier to later phases in the task were weakest in the CS-IGT, despite a lack of ceiling effects.

## Discussion

The current study is the first to incorporate a novel, social variant of the well-established Iowa Gambling Task (i.e., the S-IGT) to assess differences between younger and older adults in trust-related decision-making and learning. This task uses faces that were independently rated on high and low perceived trustworthiness^14^ to represent different decks under conditions of facial cue congruence (CS-IGT) and incongruence (IS-IGT). Specifically, trustworthy faces are paired with advantageous decks in the CS-IGT and disadvantageous decks in the IS-IGT, and vice versa for untrustworthy faces. Extending previous work, the present study examined face bias related to trustworthiness in initial choices in the S-IGT and applied hypothesis-driven analyses to investigate age-group differences in decision-making and learning under social and non-social conditions.

In summary, our results revealed that participants initially drew from decks that displayed a trustworthy over an untrustworthy face. This initial face bias was comparable across younger and older adults. For non-social learning, younger adults outperformed older adults in the IGT overall and over time, consistent with previous work^43,44^. In contrast, when drawing from decks with congruent facial cues (i.e., trustworthy faces with advantageous outcomes, untrustworthy faces with disadvantageous outcomes), both age groups had high, though not ceiling-level, performance. However, congruent cues also led to less improvement throughout the task for both age groups compared to the other task conditions (i.e., IGT and IS-IGT). Thus, the benefit of congruent facial cues on deck selection was consistent across blocks and age groups, with little evidence of learning in this condition. Critically, when drawing from decks with incongruent facial cues (i.e., untrustworthy faces paired with advantageous outcomes, trustworthy faces paired with disadvantageous outcomes), older compared to younger adults were relatively less capable of overriding their bias towards trustworthy faces over time, and adaptively using only choice-outcome associations to guide their decision-making. These primary findings from the study are discussed next.

Supporting *Hypothesis 1*, both younger and older adults initially preferred trustworthy over untrustworthy faces as reflected in their first deck choices. Here we found that facial trustworthiness cues biased deck choices at first and in the absence of any other information to signal potential choice outcomes. This bias towards trustworthy faces was initially beneficial and reinforced over time in the CS-IGT (*Hypothesis 4b*) but was detrimental to initial performance in the IS-IGT (*Hypothesis 4c*) when uncertainty over choice outcomes was high. This finding indicates that people prefer trustworthy-looking faces, as previously shown for both younger and older adults in the form of greater investment behavior^5,64^ as well as higher trustworthiness and approachability ratings for faces with greater perceived trustworthiness^5,24^.

Supporting *Hypothesis 2a* and *3a*, older adults performed worse than younger adults on the non-social, standard IGT overall and over time—replicating previous work using the IGT in older populations^43,44,65^. Poorer IGT performance in older adults was characterized in previous studies by more disadvantageous choices and delayed shifting towards more advantageous choices. This finding may be attributable to age-related differences in cognitive bias and strategies used during the IGT. For example, older compared to younger adults showed greater loss frequency bias, consistency in deck choices, and forgetting of recent outcomes^44^. Impairments in other cognitive functions, such as memory^43^, may also explain age-group differences in behavior.

Findings from the present study importantly contribute to a mixed literature on how IGT performance varies with age^65,66^, with some older adults behaving more adaptively than others perhaps due to interindividual differences^45,67,68^. For example, increased difficulties for some older adults in the IGT were independent of general cognitive functions such as attention, memory, and visual perception, and might be instead due to preclinical declines in the ventromedial prefrontal cortex or medial temporal lobe^65^. Future work will benefit from using additional cognitive and neuroanatomical measures to explain variability in performance and learning among older adults.

Comparing performance in the IGT to the S-IGT showed that facial trustworthiness cues interfered with participants’ ability to effectively evaluate potential choice outcomes. We expected that younger adults would outperform older adults in all task conditions over time (*Hypothesis 3a and 4a*), but to a lesser extent in the CS-IGT (*Hypothesis 3b and 4b*) as a measure of trust learning. Although we found that younger adults had better performance than older adults in all task conditions over time (i.e., Blocks 2–4), both age groups demonstrated comparable learning with the greatest improvement in the IGT. The inclusion of social information added complexity to decision-making and learning for both age groups. Breaking these findings down, in the CS-IGT, congruent facial cues initially led to more beneficial choices but less learning and accumulated rewards over time. In the IS-IGT, initial performance and learning were negatively impacted for both age groups, but especially for older adults. The implications of these findings are discussed next.

Younger and older adults sustained comparably high, though not ceiling-level, scores after their first draws and throughout the CS-IGT, supporting *Hypothesis 2b*. This was likely due to the dual presence of congruent cues of facial trustworthiness and positive outcomes throughout the CS-IGT. However, out of all task conditions, the CS-IGT showed the least improvement from the uncertainty phase to the experience phase. Thus, the presence of faces that were congruent with overall payouts did not lead to maximal gains. Instead, congruent facial trustworthiness cues may have diminished attention to and interfered with the evaluation of choice-outcome likelihoods over time. That is, though congruent cues aligned with first impressions of trustworthiness and initially facilitated performance, these cues may have led to *satisficing*^69^, a cognitive heuristic that involves the pursuit of adequate over optimal results, sparing the effort involved in decision-making. Though *satisficing* does not lead to maximizing utility, the usefulness of this strategy reflects the time and information constraints of real-world day-to-day decision-making; and illustrates how both younger and older adults may leverage cues in their environment to make *relatively beneficial* choices at a reduced cognitive cost. Indeed, older adults received a relative benefit from congruent facial cues, which—despite their apparent negative impact on learning—still led to rates of advantageous choices in the CS-IGT across trials that took a greater amount of experience to reach in the absence of facial cues (i.e., in the IGT).

Although the present findings did not demonstrate significant age-group differences in behavior throughout the CS-IGT, older adults have previously reported engaging in greater *satisficing* and less alternative searches than younger adults^70^. These decision-making strategies in older adults potentially reflect their preferences for processing limited options and information to spare effort and working memory, increased reliance on previous experience, and greater motivation for maintaining positive affect^70,71^. These age-related differences in decision-making strategies and heuristics, however, have been primarily described in the context of consumer and financial decision-making, warranting further exploration in dynamic, social environments^71,72^.

As in the CS-IGT, behavior in the IS-IGT revealed an initial bias for choosing faces perceived as trustworthy and against those perceived as untrustworthy. In contrast to the age-equivalent effects observed for the CS-IGT, an age-differential pattern was found for the IS-IGT. Specifically, although both younger and older adults initially had the lowest scores in the IS-IGT due to preferring decks with trustworthy faces leading to net losses (*Hypothesis 1*), younger adults outperformed older adults in the IS-IGT overall (*Hypothesis 2c*).

This divergent pattern between younger and older adults in response to incongruent cues of trustworthiness echoes previous findings on age-related differences in trustworthiness evaluations and trust learning. In particular, our results are in line with research showing that it can be more difficult for older adults to override initial impressions of trustworthiness^38^. Older compared to younger adults also tend to give more favorable evaluations of untrustworthy faces^24,27–29^ and are more likely to perseverate in engaging with untrustworthy partners^38^. However, this previous line of work had primarily focused on paradigms that only considered facial ratings or involved limited behavioral interactions with social partners. In contrast, the present study extends our understanding of younger and older adults’ trust-related decision-making and learning through a multi-round behavioral experiment in which social decision-making was also compared to performance in a non-social task with the same design and reinforcement schedule.

### Future directions

Older adults in the present study had difficulty integrating incongruent trust-related information, which may be due to age-related shifts in perception, motivation, attention, and/or memory for socioemotional information that make it harder to disambiguate or ignore incongruent cues. For example, older adults experience multimodal processing difficulties during incongruent emotion perception, which is related to deception detection^73^. Social decision-making is also especially taxing for older adults when negative information needs to be considered (e.g., positivity effect)^29^. Dynamic mental states, such as one’s emotional reactions to trust violations (e.g., feelings of anger; desire to punish^74^), may also affect social decision-making in an age-differential pattern.

Understanding the factors that influence age-group differences in incongruent information integration can help further characterize the vulnerabilities older adults face when they interact with “wolves in sheep’s clothing” (i.e., those who intend to deceive, exploit, or abuse) across social settings^75^. This is critical given that older adults are vulnerable to financial exploitation, fraud, and scams that are carried out in dynamic social environments (e.g., in-person, over the phone, or online) and by different social partners (e.g., strangers, close others)^4^. Future experimental manipulations that gauge motivation and perception (e.g., follow-up questions after making certain choices or receiving feedback) and analytic approaches such as computational modeling can be used to identify the cognitive strategies and biases at work in younger and older adults during trust-related decision-making and learning.

Findings from the present study are also qualified by the stimuli used in the S-IGT (i.e., white, middle-aged, male faces with neutral expressions). The S-IGT can be adapted in the future to incorporate additional facial stimuli attributes (e.g., race, age, sex, and emotion) to systematically examine their influences on trust learning across different populations. More exploration is needed on the effects of own-age, -sex, and -race biases as well as stereotype reliance on trust learning^59,76^. Further, the processing of positive and negative facial emotions can dynamically alter trustworthiness evaluations, add complexity to social decision-making, and increase cognitive load, which should be considered in future experiments^17,29^.

Future adaptations of the IGT should also consider the stability and generalizability of findings (e.g., inter-individual and inter-study variability^52^) when parameters of the original task are altered. It may be possible that variations to the IGT are more stable than variations of social decision-making tasks like the Trust Game. For example, even small changes to Trust Game parameters introduced wide variation in participants’ responses^77^. Behavior in the S-IGT in the present study was largely consistent with hypotheses, did not produce wide variation outside of the typical linear learning patterns observed in the standard IGT in other healthy populations, and closely followed experimental manipulations used in previous publications^47,48^. However, questions regarding the stability and generalizability of findings of the S-IGT and other variations of the IGT will require further validation. Additionally, comparing behavior in the S-IGT to social decision-making tasks that capture other important aspects of evaluating trustworthiness (e.g. using the Bluff/Challenge Game to understand how stereotyped beliefs about others bias trust-related behavior^78^), may be useful for our understanding of the impact of aging on trust-related decision-making and learning.

Beyond behavior, there are age-group differences in neural responses to trust-related information^24^*.* For example, older compared to younger adults recruit brain regions associated with mentalizing and memory to a greater extent during trust-related decision-making^36^. Different neurophysiological components are also involved in the dynamics of trust learning (e.g., during the processing of unfamiliar faces vs. when integrating prior experience)^7^, which may be altered in aging. While investigating neural responses during social decision-making is outside of the scope of this study, such work is critically needed to advance understanding of mechanisms that drive age-group differences in behavior.

The present study also only focused on trust-related decision-making and learning among healthy younger and older adults. Future investigations warrant exploring this phenomenon in clinical populations with cognitive impairments that impact decision-making, such as those at risk for Alzheimer’s disease and related dementias. It is also worthwhile to consider individuals experiencing other health challenges (e.g., chronic pain) and/or adverse life experiences that can impact the quality of their relationships, erode trust in people and institutions over time, and/or constitute a greater risk for exploitation (e.g., isolation, injustice, adversity, previous victimization)^79^.

## Conclusions

In conclusion, findings from the present study showed that younger and older adults preferred trustworthy over untrustworthy faces, especially when paired with advantageous outcomes, but facial cues that were congruent with experienced choice outcomes appeared to impair learning and result in *satisficing* in both age groups. In contrast to these age-equivalent effects, younger adults outperformed older adults in decisions with incongruent cues of trustworthiness; however, both groups adapted by making choices based on outcomes rather than cues over time. Finally, older adults performed worse than younger adults during non-social decision-making, consistent with prior research. Together these findings indicate that differences between younger and older adults in experience-dependent decision-making are magnified in social contexts that involve a “wolf in sheep’s clothing,” but may reflect age-related difficulties in incongruent information integration. Future investigations will benefit from identifying age-specific differences in cognitive strategies and biases during social decision-making and learning; systematically testing responses to different stimuli attributes; linking neural responses to behavior; and focusing on clinical populations with greater exploitative risk.

### Supplementary Information

Supplementary Information.

## Data Availability

Datasets generated and analyzed from the current study and accompanying code are available in the Open Science Framework repository: https://osf.io/cn8a2/?view_only=9673e33580a6436c8ed0f69c51b98152.
