# Author Correction: Age-group differences in trust-related decision-making and learning

**DOI:** 10.1038/s41598-024-55891-z

**Published:** 2024-03-01

**Authors:** Marilyn Horta, Alayna Shoenfelt, Nichole R. Lighthall, Eliany Perez, Ian Frazier, Amber Heemskerk, Tian Lin, Robert C. Wilson, Natalie C. Ebner

**Affiliations:** 1 Department of Psychology, University of Florida, Gainesville, FL USA; 2 Pain Research and Intervention Center of Excellence, University of Florida, Gainesville, FL USA; 3 Department of Psychology, University of Central Florida, Orlando, FL USA; 4 Graduate School of Applied and Professional Psychology, Rutgers University, New Brunswick, NJ USA; 5 Department of Psychology, University of Arizona, Tucson, AZ USA

Correction to: *Scientific Reports* 10.1038/s41598-023-50500-x, published online 02 January 2024

The original version of this Article contained errors.

In Figure 1, facial photographs were reproduced without permission from the copyright holder. These images have now been removed and replaced with schematic placeholders. The accompanying legend of Figure 1 has also been modified.

“Task conditions and design features. There were three task conditions: standard Iowa Gambling Task (IGT), congruent social Iowa Gambling Task (CS-IGT), and incongruent social Iowa Gambling Task (IS-IGT). In the CS-IGT, advantageous decks were paired with trustworthy faces and disadvantageous decks with untrustworthy faces. In the IS-IGT, this pairing was reversed.”

now reads:

“Task conditions and design features. There were three task conditions: Standard Iowa Gambling Task (IGT), Congruent Social Iowa Gambling Task (CS-IGT), and Incongruent Social Iowa Gambling Task (IS-IGT). In the CS-IGT, advantageous decks were paired with naturalistic photographs of trustworthy faces (placeholder depicted in green) and disadvantageous decks with untrustworthy faces (placeholder depicted in red). These photographs (not pictured here) were from the FACES database^46^. In the IS-IGT, this pairing was reversed.”

Additionally, in the Methods section, under the subheading ‘The standard and social Iowa Gambling Tasks’,

“The faces used in the CS-IGT and IS-IGT were independently rated as the two most trustworthy and two least trustworthy white, male, middle-aged faces with a neutral (non-emotional) expression in the FACES database^14,46^. The face models depicted in Fig. 1 each provided informed consent for publication of identifying images for research purposes, including online open-access^46^. Middle-aged faces were selected to avoid own-age bias in trust perception^30,59^ and neutral expressions were chosen to avoid emotion-related bias (i.e., negative emotions can be perceived as less trustworthy^14^).”

now reads:

“The faces used in the CS-IGT and IS-IGT were independently rated as the two most trustworthy and two least trustworthy white, male, middle-aged faces with a neutral (non-emotional) expression in the FACES database^14,46^. Middle-aged faces were selected to avoid own-age bias in trust perception^30,59^ and neutral expressions were chosen to avoid emotion-related bias (i.e., negative emotions can be perceived as less trustworthy^14^).”

The original Article has been corrected.

