# Supplementary material for: Age-group differences in trust-related decision-making and learning
Source: Sci Rep. 2024 Jan 2;14:68. doi: 10.1038/s41598-023-50500-x (PMC10762071; doi:10.1038/s41598-023-50500-x)
Supplement: Supplementary file 1 — Supplementary Information. [file 41598_2023_50500_MOESM1_ESM.pdf]

Supplementary Information

Age-group differences in trust-related decision-making and learning

Marilyn Horta, Alayna Shoenfelt, Nichole R. Lighthall, Eliany Perez, Ian Frazier, Amber Heemskerk, Tian Lin, Robert C. Wilson, & Natalie C. Ebner

Invalid Performance Check

We examined whether participants exclusively chose advantageous over disadvantageous decks as a measure of invalid performance consistent with previous work [1]. The proportion of advantageous deck choices ranged from 27% – 90% across the full task (blocks 1-5); with the mean proportion of advantageous deck choices being 55.44%. Thus, no participants were excluded from analysis for exclusively selecting advantageous decks.

Non-Replenishing Card Decks

Each task condition consisted of four non-replenishing decks of cards, and some participants ran out of cards by the end of the fourth block. For the disadvantageous decks, no participants ran out of cards from Deck A, but 17 participants (6.25% of full sample) ran out of cards from Deck B (n = 9 in Study 1; n = 8 in Study 2) by the end of Block 4. For the advantageous decks, 16 participants (5.88% of full sample) depleted Deck C (n = 8 in Study 1; n = 8 in Study 2) and 19 participants (6.99% of full sample) ran out of cards from Deck D (n = 7 in Study 1; n = 12 in Study 2) by the end of Block 4.

Results for Blocks 1-4 with Outliers Removed

As reported in the manuscript, no outliers were identified as exceeding +/- 3 SD from the mean total performance across blocks within each age group; however, four mean performance scores within blocks were identified as outliers. The pattern of results did not change when these outliers were excluded from the analysis, as reported below.

Full-Factorial Multilevel Model of Effects for Age Group, Task Condition, and Block (Hypotheses 2a, 2b, 2c, 3a, 3b)

Mirroring the results reported in the manuscript, the interaction between Age group and Task condition for overall performance was significant ($\chi^2(2) = 11.27$; $p < .01$). Pairwise comparisons indicated that younger, compared to older, adults performed better overall on the IGT ($z = 3.56$, $p < .001$) and the IS-IGT ($z = 4.26$, $p < .001$). However, overall performance was comparable between the two age groups in the CS-IGT ($z = -0.66$, $p = .51$). Together, these findings supported *Hypotheses 2a, 2b,* and *2c* that, while there were no significant age-group differences in overall performance for the CS-IGT, younger adults significantly outperformed older adults in the IGT and IS-IGT.

In addition, a significant interaction between Age group and Block was found ($\chi^2(3) = 13.56$; $p < .01$). Post-hoc examination of age-group differences by block showed that across all task conditions, younger and older adults performed comparably during Block 1 ($z = 0.58$, $p = .56$), but younger adults significantly outperformed older adults in Block 2 ($z = 2.38$, $p = .02$), Block 3 ($z = 3.90$, $p < .001$), and Block 4 ($z = 3.95$, $p < .001$). This provides support for *Hypothesis 3a,* that older age would be associated with less

improvement over time across all task conditions, consistent with well-established age-related impairments in learning.

In addition, the interaction between Task condition and Block was significant ($\chi^2(6) = 28.45$; $p < .001$). Post-hoc analysis revealed that Block 1 performance was significantly lower in the IS-IGT than the CS-IGT ($z = -4.94$, $p < .001$) and the IGT ($z = -2.80$, $p = .01$) across all participants. Block 4 performance was highest in the IGT compared to both the CS-IGT ($z = 3.20$, $p < .01$) and the IS-IGT ($z = 2.71$, $p = 0.01$). Block 4 performance was comparable between the CS-IGT and the IS-IGT ($z = 0.40$, $p = .69$). As discussed in the manuscript, these results suggest that, over time, participants in the IS-IGT learned to ignore the facial cues – comparable to performance levels of participants in the CS-IGT. However, learning was greatest in the IGT which did not include additional congruent or incongruent facial cues to process. Age group did not significantly interact with Task condition and Block ($\chi^2(6) = 5.75$; $p = .45$).

Results with Blocks 1-5

The analyses reported in the manuscript were conducted on the first four of five blocks due to the non-replenishing deck design for the IGT and the S-IGT. Results on the full task (Blocks 1-5) were parallel to those reported in the manuscript, however learning and performance plateaued after Block 4 likely due to the non-replenishing deck design. Full results for the main analysis with Block 5 included are reported below.

Full-Factorial Multilevel Model of Effects for Age Group, Task Condition, and Block (Hypotheses 2a, 2b, 2c, 3a, 3b)

The interaction between Age group and Task condition was significant ($\chi^2(2)$ = 10.82; p = .005). Pairwise comparisons indicated that younger, compared to older, participants performed better overall on the IGT (z = 4.13, p < .001) and the IS-IGT (z = 4.88, p < .001). However, overall performance was comparable between the two age groups in the CS-IGT (z = 0.18, p = .86). As reported in the main manuscript, these findings supported *Hypotheses 2a*, *2b*, and *2c* that while younger and older adults performed comparably on the CS-IGT, younger participants significantly outperformed older participants in the IGT and the IS-IGT.

There was also a significant interaction between Age group and Block ($\chi^2(4)$ = 10.03; p = .04). Post-hoc examination of age-group differences by block showed that across all tasks, younger and older adults performed comparably during Block 1 (z = 0.38, p = .71), but younger adults significantly outperformed older adults in Block 2 (z = 2.21, p = .03), Block 3 (z = 3.58, p < .01), Block 4 (z = 3.58, p < .001), and Block 5 (z = 3.81, p < .001). This again provides support for *Hypothesis 3a*, that older age would be associated with less improvement over time across all task conditions, consistent with well-established age-related impairments in learning.

In addition, the interaction between Task condition and Block was significant ($\chi^2(8)$ = 26.38; p < .001). Post-hoc examinations revealed that Block 1 performance was significantly lower in the IS-IGT than the CS-IGT (z = -4.51, p < .001) and IGT (z = -2.56, p = .01) across all participants. Block 4 performance was highest in the IGT compared to both the CS-IGT (z = 2.76, p < .01) and the IS-IGT (z = 2.30, p = 0.02). Block 4 performance was comparable between the CS-IGT and the IS-IGT (z = 0.41, p = .69). In

Block 5, performance in the IGT was greater than performance in the CS-IGT (z = 2.53, p = .01) but not in the IS-IGT (z = 1.53, p = .13). There was no difference in performance between the CS-IGT and the IS-IGT (z = -1.16, p = .25). These findings suggest that learning was greatest in the non-social IGT, but performance plateaued after Block 4. Age group again did not significantly interact with Task condition and Block ($\chi^2(8)$ = 8.24; p = .41).

Supplementary Table 1. Design Factors for Each Hypothesis and Statistical Test

**Hypothesis**	**Statistical Test**	**Design**	**Within-subjects Factor**	**Between-subjects Factor**	**Outcome Variable**
H1	Chi-square test	2 Face (trustworthy vs. untrustworthy)	Face	N/A	Percentage of first deck choice (trustworthy face vs. untrustworthy face)
H1	Cochran-Mantel-Haenszel test	2 Age group (younger vs. older) x 2 Face (trustworthy vs. untrustworthy)	Face	Age group	Percentage of first deck choice (trustworthy face vs. untrustworthy face)
H2a, H2b, H2c, H3a, H3b	Multilevel modeling	2 Age group (younger vs. older) x 3 Task condition (IGT vs. CS-IGT vs. IS-IGT) x 4 Block (four 20-trial blocks)	Block	Age group, Task condition	Performance (i.e., total number of draws from advantageous decks minus total number of draws from disadvantageous decks, across four 20-trial blocks)
H4a, H4b, H4c	Multilevel modeling	2 Age group (younger vs. older) x 3 Task condition (IGT vs. CS-IGT vs. IS-IGT) x 2 Choice phase (first 40 trials [uncertainty] vs. last 40 trials [experience])	Choice phase	Age group, Task condition	Performance (i.e., total number of draws from advantageous decks minus total number of draws from disadvantageous decks, across four 20-trial blocks)

Note. All analyses controlled for Data source (Study 1 vs. Study 2) where applicable.

Supplementary Table 2. Main effects and interactions of the first full-factorial multilevel model; with Age group (between-subject: younger vs. older) X Task condition (between-subject: IGT vs. CS-IGT vs. IS-IGT) X Block (within-subject: four 20-trial blocks) as independent variables and Performance (i.e., number of advantageous deck selections minus number of disadvantageous deck selections for each of 4 blocks) as outcome variable.

Hypothesis	Main Effects/Interactions	Wald's χ2 Test Statistic (df)	p-value
	Age group	13.29 (1)	.003
	Task condition	15.61 (2)	< .001
	Block	77.78 (3)	< .001
H2a, H2b, H2c	Age group X Task condition	10.68 (2)	.005
H3a	Age group X Block	9.62 (3)	.02
H3b	Task condition X Block	24.14 (6)	< .001
H3a, H3b	Age group X Task condition X Block	3.82 (6)	.701

Supplementary Table 3. Main effects and interactions of the second full-factorial multilevel model; with Age group (between-subject: younger vs. older) X Task condition (between-subject: IGT vs. CS-IGT vs. IS-IGT) X Choice phase (within-subject: first 40 trials [uncertainty phase] vs. last 40 trials [experience phase]) as independent variables and Performance (i.e., number of advantageous deck selections minus number of disadvantageous deck selections computed for each phase) as outcome variable.

**Hypothesis**	**Main Effects/Interactions**	**Wald's χ^2 Test Statistic (df)**	**p-value**
	Age group	13.19 (1)	< .001
	Task condition	15.64 (2)	< .001
	Choice phase	62.75 (1)	< .001
	Age group X Task condition	10.75 (2)	.005
H4a	Age group X Choice phase	6.75 (1)	.009
H4b, H4c	Task condition X Choice phase	13.13 (2)	.001
	Age group X Task condition X Choice phase	2.35 (2)	.309

References

1. Gansler DA, Jerram MW, Vannorsdall TD, Schretlen DJ. Comparing alternative metrics to assess performance on the Iowa Gambling Task. Journal of Clinical and Experimental Neuropsychology. 2011 Nov 1;33(9):1040–8.
